# Preclinical pharmacokinetic evaluation to facilitate repurposing of tyrosine kinase inhibitors nilotinib and imatinib as antiviral agents

**DOI:** 10.1186/s40360-018-0270-x

**Published:** 2018-12-04

**Authors:** Hari Krishna Ananthula, Scott Parker, Erin Touchette, R. Mark Buller, Gopi Patel, Daniel Kalman, Johanna S. Salzer, Nadia Gallardo-Romero, Victoria Olson, Inger K. Damon, Tessa Moir-Savitz, Larry Sallans, Milton H. Werner, Catherine M. Sherwin, Pankaj B. Desai

**Affiliations:** 1 James L. Winkle College of Pharmacy, University of Cincinnati, Cincinnati, OH USA; 2 Department of Molecular Microbiology and Immunology, School of Medicine, Saint Louis University, St. Louis, MO USA; 3 Department of Pathology and Laboratory Medicine, Emory University School of Medicine, Atlanta, GA USA; 4 Centers for Disease Control and Prevention, Atlanta, GA USA; 5 Battelle Memorial Institute, Columbus, OH USA; 6 Mass Spectrometry Facility, University of Cincinnati, Cincinnati, OH USA; 7 Inhibikase Therapeutics, Inc., Atlanta, GA USA; 8 Division Clinical Pharmacology, University of Utah School of Medicine, Salt Lake City, UT USA

**Keywords:** Tyrosine kinase inhibitor, Pharmacokinetics, Allometry, Animal rule

## Abstract

**Background:**

Several tyrosine kinase inhibitors (TKIs) developed as anti-cancer drugs, also have anti-viral activity due to their ability to disrupt productive replication and dissemination in infected cells. Consequently, such drugs are attractive candidates for “repurposing” as anti-viral agents. However, clinical evaluation of therapeutics against infectious agents associated with high mortality, but low or infrequent incidence, is often unfeasible. The United States Food and Drug Administration formulated the “Animal Rule” to facilitate use of validated animal models for conducting anti-viral efficacy studies.

**Methods:**

To enable such efficacy studies of two clinically approved TKIs, nilotinib, and imatinib, we first conducted comprehensive pharmacokinetic (PK) studies in relevant rodent and non-rodent animal models. PK of these agents following intravenous and oral dosing were evaluated in C57BL/6 mice, prairie dogs, guinea pigs and Cynomolgus monkeys. Plasma samples were analyzed using an LC-MS/MS method. Secondarily, we evaluated the utility of allometry-based inter-species scaling derived from previously published data to predict the PK parameters, systemic clearance (CL) and the steady state volume of distribution (Vss) of these two drugs in prairie dogs, an animal model not tested thus far.

**Results:**

Marked inter-species variability in PK parameters and resulting oral bioavailability was observed. In general, elimination half-lives of these agents in mice and guinea pigs were much shorter (1–3 h) relative to those in larger species such as prairie dogs and monkeys. The longer nilotinib elimination half-life in prairie dogs (i.v., 6.5 h and oral, 7.5 h), facilitated multiple dosing PK and safety assessment. The allometry-based predicted values of the Vss and CL were within 2.0 and 2.5-fold, respectively, of the observed values.

**Conclusions:**

Our results suggest that prairie dogs and monkeys may be suitable rodent and non-rodent species to perform further efficacy testing of these TKIs against orthopoxvirus infections. The use of rodent models such as C57BL/6 mice and guinea pigs for assessing pre-clinical anti-viral efficacy of these two TKIs may be limited due to short elimination and/or low oral bioavailability. Allometry-based correlations, derived from existing literature data, may provide initial estimates, which may serve as a useful guide for pre-clinical PK studies in untested animal models.

## Background

Recent reports suggest that tyrosine kinase inhibitors (TKIs), which are extensively used as targeted anti-cancer agents, may also have anti-viral applications. As a consequence of their ability to inhibit the activity of cellular Abelson tyrosine kinases (c-Abl1 and c-Abl2), viral egress from infected cells is impeded preventing further spread of disease [1–3]. Accordingly, these agents are being investigated to assess their efficacy against viral pathogens such as monkeypox virus, variola virus (the causative agent of smallpox), and filoviruses (Ebola and Marburg). The potential use of such agents as anti-viral therapeutics represents an attractive strategy for repositioning drugs approved by US Food and Drug Administration (FDA) as readily available medical countermeasures (MCMs) against such biological threats. Given that the therapeutic window and critical aspects of the clinical pharmacology of such compounds are well delineated, these agents can be readily deployed if efficacy can be established and regulatory approval is achieved.

A major challenge in the development of effective therapeutics against highly pathogenic viral diseases is the ethical constraint that prohibits human trials and the pragmatic issues associated with conducting field efficacy studies during a sporadic outbreak and identifying asymptomatic patients who might benefit from therapy [4]. In these situations, efficacy assessments require the use of appropriate pre-clinical approaches that employ both in vitro assays and animal models, which are best suited for viral replication and recapitulate human disease. Animal models provide insights beyond what can be gained from in vitro evaluation of the antiviral activity. An ideal model is one which utilizes a human equivalent infectious dose and a route of infection that mimics natural transmission of the pathogen and exhibits a disease course, morbidity, and mortality similar to human disease [5]. To provide a regulatory framework for this purpose, the FDA devised the “Animal Efficacy Rule” (a.k.a ‘Animal Rule’), directing the use of appropriate animal models to demonstrate the effectiveness of MCMs [4].

A critical issue, however, is that PK information on test agents is not routinely available in the specific animal models necessary for evaluating efficacy against pathogens. Thus, an important prerequisite is to determine key PK parameters of test agents in these animal species so anti-viral effectiveness can be assessed with dosing regimens likely to yield plasma drug levels within the established therapeutic range. Eventually, such studies can then help derive pharmacokinetic-pharmacodynamic (PK-PD) correlations so that appropriate doses may be employed to yield the systemic exposure necessary of anti-viral activity in humans.

Some of the animal models that are used for anti-viral testing include susceptible strains of mice, guinea pigs, prairie dogs and monkeys [6, 7]. Due to their sensitivity to most inoculation routes, mice have been widely used to study various pathogens. In the case of monkeypox virus, prairie dog has been shown to be a suitable animal model [8]. For instance, the efficacy of oral administration of ST-246 against a lethal respiratory challenge with monkeypox virus was tested in prairie dogs [9]. Finally, non-human primates have also been used to evaluate antivirals against orthopoxviruses, particularly monkeypox virus and variola virus [10, 11]. Efficacy of several investigational agents against filoviruses has also been carried out in guinea pigs, and non-human primates with the postulation as basic disease manifestation are similar to that seen in humans [7, 12].

Efforts are currently in progress to investigate the use of nilotinib and imatinib, two marketed TKIs, as antiviral agents employing the above-indicated animal species. As an important first step, we evaluated the PK and absolute oral bioavailability of these agents in mice, prairie dogs, guinea pigs and Cynomolgus monkeys. The primary objective was to use these results to optimize the dosing regimen to attain a systemic exposure within the clinical therapeutic range to facilitate efficacy testing against the challenge virus. Secondarily, we assessed the utility of allometry-based inter-species PK modeling as a predictive tool for PK parameters including clearance and volume of distribution in animal species such as prairie dogs typically not used in pre-clinical drug development stages.

## Methods

### Materials

Nilotinib and imatinib were purchased from Selleck Chemicals (Houston, TX). HPMC (hydroxypropyl methylcellulose, a.k.a. Methocel E6) was provided as a research sample from Dupont Chemicals and Kolliphor® EL (a.k.a. Cremophor EL; polyoxyl castor oil) was purchased from Sigma-Aldrich. Ethyl acetate, methanol, acetonitrile and all other analytical grade reagents were purchased from Fisher Scientific.

### Formulations

Imatinib was formulated as a solution in sterile water for both intravenous (IV) and oral administration in all species. Nilotinib was formulated in ethanol: PEG300: Kolliphor EL (1.5:4.5:20, *v*/v/v) in 3.7% dextrose solution for intravenous administration in all species. For oral administration, nilotinib was formulated as nilotinib/NMP (1-methyl-2-pyrrolidinone) (20 mg/ml) in PEG 300 (1:10) for mice and initial prairie dog studies. For oral administration in guinea pigs, monkeys and subsequent studies in prairie dogs, nilotinib was formulated as a suspension consisting of 1.5% Avicel®-RC 591 and 0.3% HPMC.

### Animal studies

PK studies in animals were approved by the Institutional Animal Care and Use Committee (IACUC) of the institution performing the study. Mouse studies were conducted at Emory University (C57BL/6, IACUC # 2003021). C57BL/6 mice (20 g) were obtained from Jackson Laboratory. Twenty-four animals were used for each route of administration and both the genders were randomly included (*n* = 3 mice per time point). The intravenous dose was administered via tail vein injection at an injection volume of 5 ml/kg, and the oral dose was given via gavage at a dose volume of 10 ml/kg. The blood samples (0.5–1 ml) were withdrawn from the submandibular vein and collected into EDTA tubes at pre-dose and at 0.5, 1, 2, 4, 8, 12 and 24-h post-dose. Mice were sacrificed with carbon dioxide asphyxiation following bleeds. Plasma was prepared and stored at -80^o^ C until bioanalysis.

Prairie dog PK studies were performed at Centers for Disease Control and Prevention (CDC, Atlanta). Twenty-six wild-caught male black-tailed prairie dogs (*Cynomys ludovicianus*) aged 1–2 years were used in this study in accordance with CDC IACUC policies and procedures under an approved animal protocol (IACUC # 2450SALPRAC). The prairie dogs were obtained from a provider regulated and licensed by United States Department of Agriculture (dealer’s license number: 74-B-0638 and wildlife permit number 6523). The animals were collected in Lubbock, TX. All animals are given full physical examination by a veterinarian prior to being shipped to CDC. Prairie dogs were individually housed for the 24-h period for each study. The animals received a single oral dose of nilotinib prepared as either NMP/PEG 300 formulation (*n* = 5) or Avicel/HPMC formulation (*n* = 6) or intravenous dose (*n* = 5). Another set of animals received a single oral dose (*n* = 5) or intravenous dose (*n* = 5) of imatinib. Serial blood samples (~ 200–400 μl) were taken pre-dose and at 0.5, 1, 2, 4, 8, 12 and 24 h following oral administration at a dose volume of 2 ml/kg or intravenous administration at an injection volume of 1 ml/kg. For each blood sample collection, prairie dogs were anesthetized with 5% isoflurane gas and maintained with 1–3% isoflurane during sample collection through peripheral veins. Plasma was prepared and stored at -70^o^ C until bioanalysis. Additionally, multiple dose PK study of nilotinib was performed in prairie dogs at three different dosage regimens, 7 mg twice-daily, 20 mg once-daily, and 20 mg twice-daily for 7 days using NMP/PEG 300 formulation. Blood samples were collected immediately after nilotinib administration on Days 1 and 7 (to represent peak drug levels) and pre-dose sample on Day 7 to reflect steady-state trough drug level, during the seven-day drug administration.

PK study of nilotinib in guinea pigs upon intravenous or oral routes was conducted at University of Cincinnati (IACUC # 13–09–03-01). Male Hartley guinea pigs (450–650 g) were procured from Charles River. Nine animals were used, three (*n* = 3) for each route of administration. The intravenous dose was given via jugular vein cannula, and the oral dose was given via gavage. The volume of dose administration was 1 mg/kg for both routes of administration. Blood samples (200–250 μl) were collected by serial sampling through saphenous or femoral veins into EDTA tubes at pre-dose and at 0.25, 0.5, 1, 2, 4, 8, 12 and 24 h after administration. Additional samples were collected at 0.033 and 0.083 h after intravenous dosing. Plasma was prepared and stored at -80^o^ C until bioanalysis.

The oral and intravenous PK study of nilotinib in Cynomolgus monkeys was performed at Battelle Memorial Institute, Columbus, Ohio (IACUC # 38020). Six Animals (3.5 kg) were procured from Charles River, three (*n* = 3) for each route of administration. Animals were fasted overnight before dosing and at least 1 h following dose administration. The intravenous dose was given via a saphenous vein at an injection volume of 1 ml/kg, and oral dosing was performed via gavage at a dose volume of 5 ml/kg. The blood samples (~ 1 ml) were collected through saphenous or femoral veins into tubes containing K_2_·EDTA at pre-dose and at approximately 0.083, 0.25, 0.5, 1, 2, 4, 8, 12 and 24 h. post-dose. Plasma was prepared and stored at-70^o^ C until bioanalysis.

### Bioanalysis

Sample preparation and bioanalysis was performed at the University of Cincinnati. For extraction, 50 μl of plasma samples were transferred to glass tubes. Plasma samples containing nilotinib were first acidified with 10 μl formic acid. Subsequently, 10 μl of internal standard was added (d^3^-nilotinib or d^8^-imatinib) to the samples and mixed. Ethyl acetate and methylene chloride (1000 μl) were employed as extraction solvents for nilotinib and imatinib, respectively. The extraction solvent was separated by centrifugation. For nilotinib samples, 800 μl of the supernatant organic fraction was collected. For imatinib samples, 800 μl of the bottom organic layer was collected. Collected fractions were evaporated using centrifugal evaporator. Nilotinib samples were then reconstituted in 100 μl acetonitrile containing 0.2% formic acid, and imatinib samples were reconstituted in 100 μl methanol: water (60%:40%).

Analysis of extracted samples was performed by an LC-MS/MS method. For imatinib, mobile phase consisted of an isocratic solvent: 71.75%: 15.00%: 13.25% (water: methanol: acetonitrile) containing 0.2% formic acid. For nilotinib, mobile phase consisted of a 30%:70% solution of acetonitrile with 0.2% formic acid and 10 mM ammonium formate with 0.2% formic acid. The column was Synergi™ 4 μm Polar-RP 50 × 2.00 mm (Phenomenex) run at a flow rate of 400 μl/min with an injection volume of 5 μl (partial loop). The retention time was 6.2 mins for nilotinib and 2.3 mins for imatinib. The analysis was performed using a Thermo Scientific LTQ-FT™ mass spectrometer operated in positive-ion electrospray mode. The source voltage was held at 5 kV, with a capillary temperature of 275 °C. The product ion scans were acquired in profile mode using an isolation width of 2 and a normalized collision energy of 20 for nilotinib and 25 for imatinib. The following ion chromatograms were acquired and quantified: for nilotinib, the *m/z* 530 parent ion producing the *m/z* 289 product ion; for d^3^-nilotinib (internal standard), the *m/z* 533 parent producing the *m/z* 289 product ion; for imatinib, the *m/z* 494 parent ion producing the *m/z* 394 product ion; for d^8^-imatinib (internal standard), the *m/z* 502 parent producing the *m/z* 394 product ion. The calibration curves ranging from 10 ng/ml to 10 μg/ml were generated from plasma-extracted standards immediately preceding and following the sequence of samples. A comparison between the two curves ensured experimental integrity.

### Pharmacokinetic analysis and interspecies correlation

Pharmacokinetic analysis was performed on either mean plasma concentration-time data (mouse) or on the individual plasma concentration-time data (prairie dog, guinea pig, and monkey) employing Phoenix® WinNonlin 6.4®_._ PK parameters such as maximum plasma concentration (C_max_), the time corresponding to C_max_ (T_max_), terminal half-life (T_1/2_), the volume of distribution (V_d_) and clearance (CL) were calculated by non-compartmental methods and presented as the arithmetic mean ± standard deviation (SD). The bioavailability (*F*) was estimated by dividing the mean dose-normalized area under the plasma concentration-time curve from time 0 extrapolated to infinity (AUC_inf_) upon oral dose by the mean dose-normalized AUC_inf_ upon intravenous dose.

Allometric correlation between body weight (BW) and CL or volume of distribution at steady state (V_ss_) was investigated as a first step followed by a prediction of CL and V_ss_ in prairie dogs. Intravenous PK parameters previously reported in literature in mice, monkeys, rats and beagle dogs [13] were used for allometric correlation of nilotinib. For imatinib, PK parameters reported in mouse [14], rats [15], rhesus monkeys [16] and beagle dogs [17] were used. Pharmacokinetic information from literature enabled allometric correlation to predict PK parameters in prairie dogs. The following allometric methods were investigated for CL prediction. Simple allometry (SA) (Eq. 1); SA with fu_p_ (fraction unbound in plasma) correction (Eq. 2) and rule of exponents (ROE) (Eqs. 3 or 4). Based on the ROE, if exponent (b) is within 0.55 to 0.70, SA without any correction was used to predict CL. If, b ≥ 0.71 and < 1, CL was corrected by maximum lifespan potential (MLP, Eq. 5) for each species and the allometric correlation was performed between CL × MLP vs. BW to predict CL. If, b ≥ 1 and < 1.3, CL was corrected by brain weight (BrW) for each species and the allometric correlation was performed between CL × BrW vs. BW to predict CL. The following allometric methods were investigated for V_ss_ prediction. SA-V_ss_ (Eq. 6); SA-fu_p_-V_ss_ (Eq. 7); The detailed explanation of all these methods was reported earlier by the PhRMA CPCDC initiative on predictive models of human PK prediction [18]. The protein binding of nilotinib was reported to be greater than 97% (fu_p_ ranged from 0.009 to 0.026) within all the preclinical species and humans [13]. Likewise, protein binding of imatinib was between 81 to 97% in preclinical species and humans [16, 19]. The following equations describe the allometric correlations.

1 $$ \mathrm{CL}=\mathrm{a}\times \mathrm{B}{\mathrm{W}}^{\mathrm{b}} $$

2 $$ \frac{\mathrm{CL}}{{\mathrm{fu}}_{\mathrm{P}}}=\mathrm{a}\times {\mathrm{BW}}^{\mathrm{b}} $$

3 $$ \mathrm{CL}\times \mathrm{MLP}\ \mathrm{or}\ \mathrm{CL}\times \mathrm{B}\mathrm{rW}=\mathrm{a}\times \mathrm{B}{\mathrm{W}}^{\mathrm{b}} $$

4 $$ \frac{\mathrm{CL}}{{\mathrm{fu}}_{\mathrm{P}}}\times \mathrm{MLP}\ \mathrm{or}\frac{\mathrm{CL}}{{\mathrm{fu}}_{\mathrm{P}}}\times \mathrm{B}\mathrm{rW}=\mathrm{a}\times \mathrm{B}{\mathrm{W}}^{\mathrm{b}} $$

5 $$ \mathrm{MLP}=10.839\times {\mathrm{BrW}}^{0.636}\times {\mathrm{BW}}^{-0.225} $$

6 $$ {\mathrm{V}}_{\mathrm{ss}}=\mathrm{a}\times \mathrm{B}{\mathrm{W}}^{\mathrm{b}} $$

7 $$ \frac{{\mathrm{V}}_{\mathrm{ss}}}{{\mathrm{fu}}_{\mathrm{P}}}=\mathrm{a}\times \mathrm{B}{\mathrm{W}}^{\mathrm{b}} $$

## Results

### Pharmacokinetics of nilotinib

**Fig. 1:**
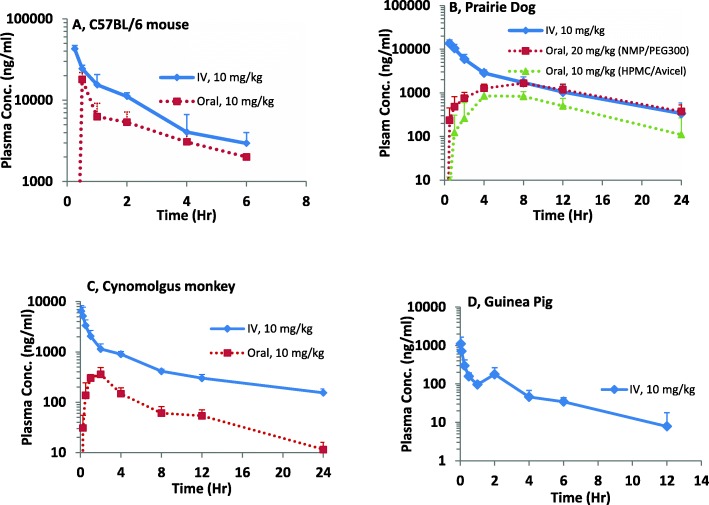
Plasma Concentration-time plots of nilotinib in (**a**) C57BL/6 mice, (**b**) prairie dogs, (**c**) monkeys, (**d**) guinea pigs after a single intravenous or oral dose. Solid line represents IV administration and dotted line represents oral administration. Oral PK profile in guinea pigs was not presented as the plasma levels were below the lower limit of quantification

The plasma concentration-time profiles of nilotinib in C57BL/6 mice, prairie dogs, guinea pigs and monkeys are plotted in semilog scale in Fig. 1. The PK parameters calculated from measured nilotinib plasma levels after a single intravenous or oral dose are summarized in Table 1.

**Table 1:** Summary of preclinical PK parameters of nilotinib after a single intravenous or oral dose in preclinical species. Data, mean ± SD

PK parameter	C57BL/6 mice	Prairie dogs		Cynomolgus monkeys
IV Dose (mg/kg)	10	10		10
No. of animals	3^a^	5		3
T^1/2^ (h)	1.81	6.51 ± 2.97		7.79 ± 0.71
MRT (h)	2.21	6.7 ± 2.9		8.9 ± 1.1
CL (ml/hr./kg)	131.14	190 ± 77		639 ± 141
V_ss_ (ml/kg)	289.77	1157 ± 326		5737 ± 1783
AUC_0-inf_ (ng.hr./ml)	76,252	57,551 ± 15,508		16,135 ± 3296
Oral Dose (mg/kg)	10^b^	20^b^	10^c^	10^c^
No. of animals	3^a^	5	6	3
T_max_ (h)	0.50	7.2 ± 1.79	5.6 ± 2.19	1.67 ± 0.58
C_max_ (ng/ml)	17,979	1673 ± 315	951 ± 255	410 ± 46
Apparent T^1/2^ (h)	2.94	7.57 ± 2.01	3.5 ± 0.6	5.16 ± 0.52
AUC_0-inf_ (ng.hr./ml)	38,366	27,991 ± 6842	9329 ± 3630	2103 ± 468
Bioavailability (%)	50	24	16	13

^a^per time point; ^b^oral dose prepared in NMP/PEG 300; ^c^oral dose prepared in Avicel/HPMC

**Fig. 2:**
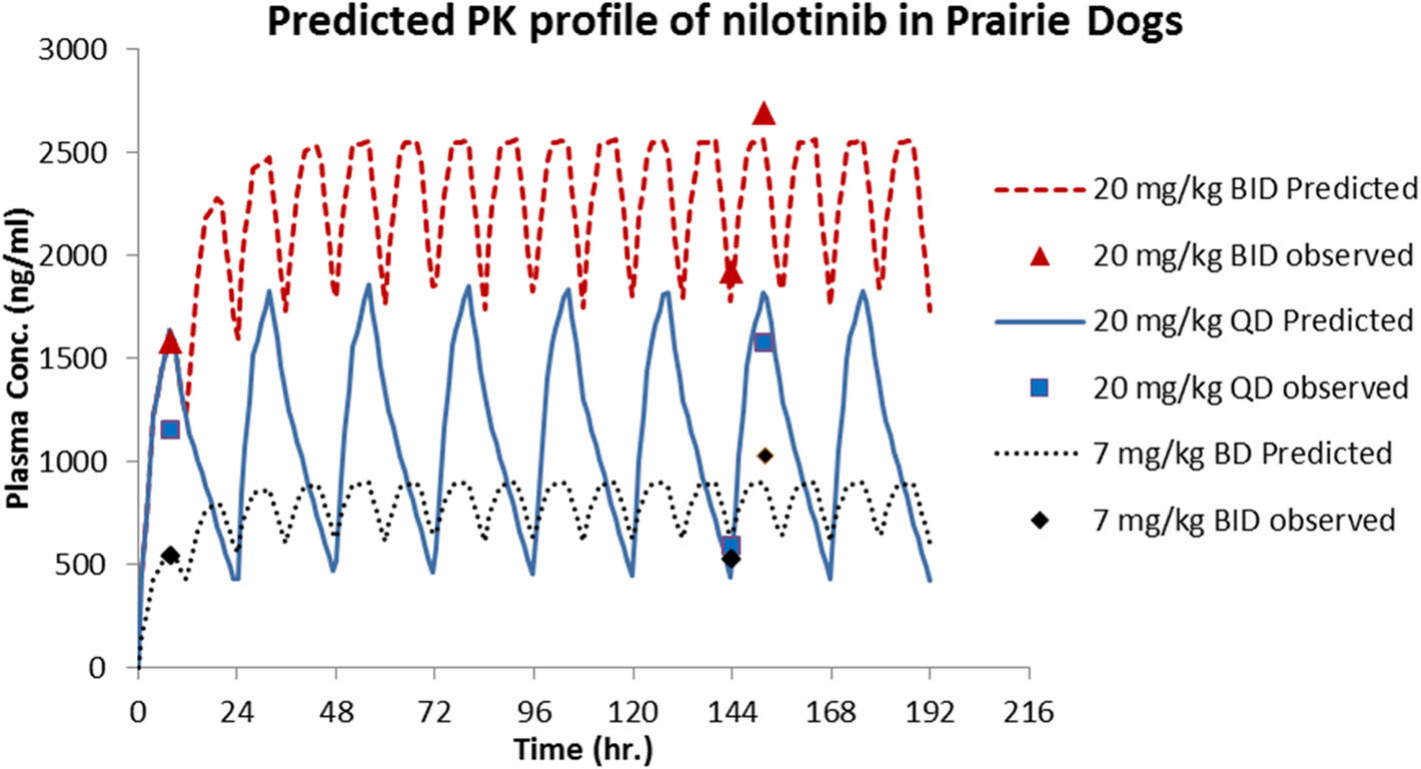
Prediction of nilotinib peak and trough plasma levels in prairie dogs upon multiple dosing. The dose groups include 7 mg/kg twice-daily, 20 mg/kg once-daily and 20 mg/kg twice-daily. Sold or dotted lines represent predicted profiles and the dots represented observed data

In C57BL/6 mice (*n* = 3 per time point), the oral terminal half-life of nilotinib was 2.94 h. With a 10 mg/kg oral dose, the C_max_ of around 18 μg/ml was achieved in 30 min after dosing. Oral bioavailability in C57BL/6 mice was 50%. In prairie dogs (*n* = 5) administered a 20 mg/kg oral dose of nilotinib formulated in NMP and PEG 300, a longer terminal half-life of 7.57 h was observed, which was similar to half-life upon 10 mg/kg intravenous dose. The drug absorption was delayed with an average peak plasma concentration of 1673 ng/ml appearing 7.2 h post-dose. Further, there was large variability in plasma concentrations between animals at all time points with a coefficient of variation (% CV) ranging from 18 to 91%. The absolute oral bioavailability was low, approximately 24%. Based on this single dose PK data, we recommended employing three dosing regimens, 7 mg twice-daily, 20 mg once-daily and 20 mg twice-daily, to evaluate multiple dose tolerance and determine steady-state plasma nilotinib levels. The previously obtained single dose PK data were used for predicting steady-state drug levels following multiple dose administration using the principle of superposition. As part of the multiple dose study, nilotinib plasma levels were measured at the time points corresponding to peak levels on days 1 and 7 and pre-dose level on day 7, which corresponds to steady state trough level. The predicted multiple dosing profiles for the three dosing regimens and the observed plasma concentration data are shown in Fig. 2. Overall, the predicted peak and trough levels on Days 1 and 7 are within the ±25% of reported levels seen upon each dosage regimen. Our approach facilitated prediction of plasma nilotinib levels in prairie dogs upon multiple doses, using prior knowledge of single dose PK profile. When nilotinib was given orally (*n* = 6) at 10 mg/kg dose formulated in Avicel/HPMC as a suspension, a terminal half-life of 3.5 h was observed. The bioavailability of nilotinib suspension formulation was found to be 16%.

In guinea pigs (*n* = 3), nilotinib extensively distributed into tissues with a V_z_ of 37.64 L/kg followed by high CL (11.9 L/hr./kg). Elimination half-life was short (2.1 h), and systemic nilotinib levels rapidly declined within 1 h of 10 mg/kg intravenous administration. Upon 10 mg/kg oral dose in guinea pigs, nilotinib plasma concentrations were found to be below 10 ng/ml at all the sampling times.

In Cynomolgus monkeys (*n* = 3), the oral terminal half-life was found to be 5.16 h. A maximum plasma level (mean C_max_) of 410 ng/ml was observed at 1.67 h (mean T_max_) upon 10 mg/kg oral dose. Drug absorption was incomplete with an absolute oral bioavailability estimated as 13%.

### Pharmacokinetics of imatinib

**Fig. 3:**
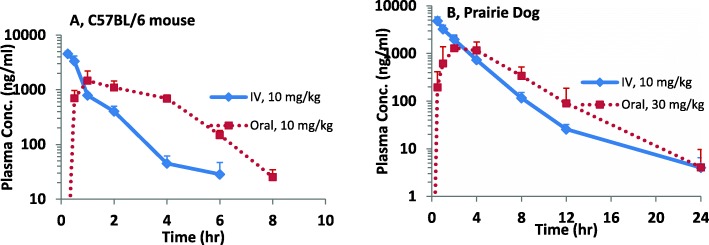
Plasma Concentration-time plots of imatinib in (**a**) C57BL/6 mice, (**b**) prairie dogs after a single intravenous or oral dose. Solid line represents IV administration and dotted line represents oral administration

PK of imatinib was investigated in C57BL/6 mice and prairie dogs. The plasma concentration-time profiles of imatinib are shown in Fig. 3. PK parameters calculated from measured imatinib plasma levels after a single intravenous or oral dose are indicated in Table 2. C57BL/6 mice exhibited complete imatinib absorption with a maximum plasma concentration of 1468 ng/ml achieved 1 h after the 10 mg/kg oral dose. The half-life of imatinib was 0.84 h. In prairie dogs, upon 30 mg/kg dose, the oral terminal half-life was 2.2 h (*n* = 4) and was similar to intravenous route (*n* = 5). A maximum plasma concentration (C_max_) of 1677 ng/ml was achieved, 3 h after the drug administration. The plasma levels were highly variable between prairie dogs with high % CV (greater than 50%) at all time points. One animal was excluded from PK analysis due to relatively low drug levels and much longer T_max_ of 12 h upon oral dose. Overall, imatinib oral bioavailability value in prairie dogs was low (~ 22%).

**Table 2:** Summary of preclinical PK parameters of imatinib (mean) after a single intravenous or oral dose in preclinical species. Data, mean values

PK parameter	C57BL/6 mice	Prairie dogs
IV Dose (mg/kg)	10	10
No. of animals	3^a^	5
T^1/2^ (h)	0.88	2.8 ± 1
MRT (h)	0.77	2.02 ± 0.24
CL (ml/hr./kg)	2212	821 ± 179
V_ss_ (ml/kg)	1697	1666 ± 464
AUC_0-inf_ (ng.hr./ml)	4522	12,558 ± 2189
Oral Dose (mg/kg)	10	30
No. of animals	3^a^	4
T_max_ (h)	1	3 ± 1.15
C_max_ (ng/ml)	1468	1677 ± 834
Apparent T^1/2^ (h)	0.84	2.2 ± 0.6
AUC_0-inf_ (ng.hr./ml)	4852	8092 ± 3012
Bioavailability (%)	107	22

^a^per time point

### Prediction of clearance and volume of distribution in prairie dogs

**Fig. 4:**
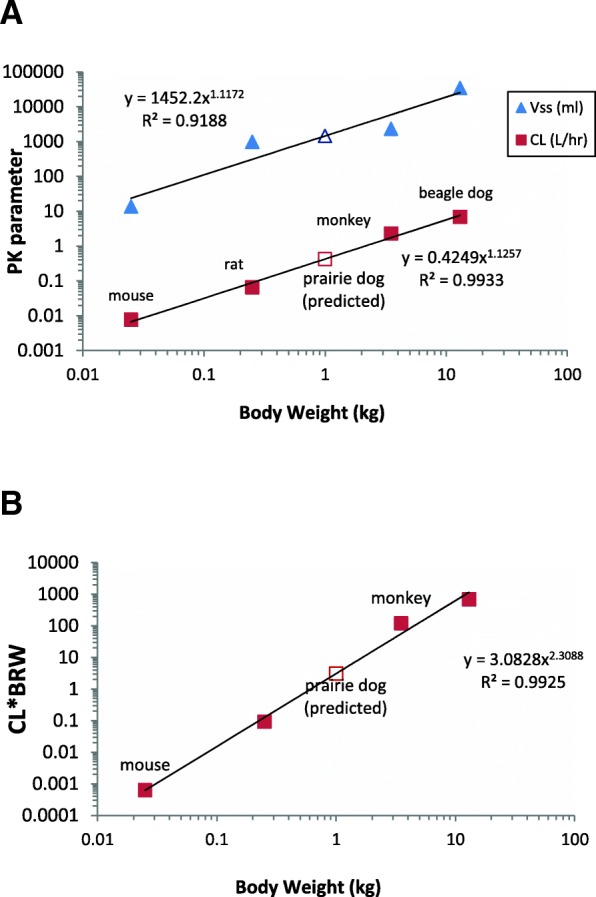
Allometric correlation plots of nilotinib (**a**) Simple allometry, (**b**) Simple allometry with ROE correction. The solid triangle symbol (▲) represents observed volume of distribution and solid square symbol (■) represents observed clearance. The open symbols represent predicted values.

**Fig. 5:**
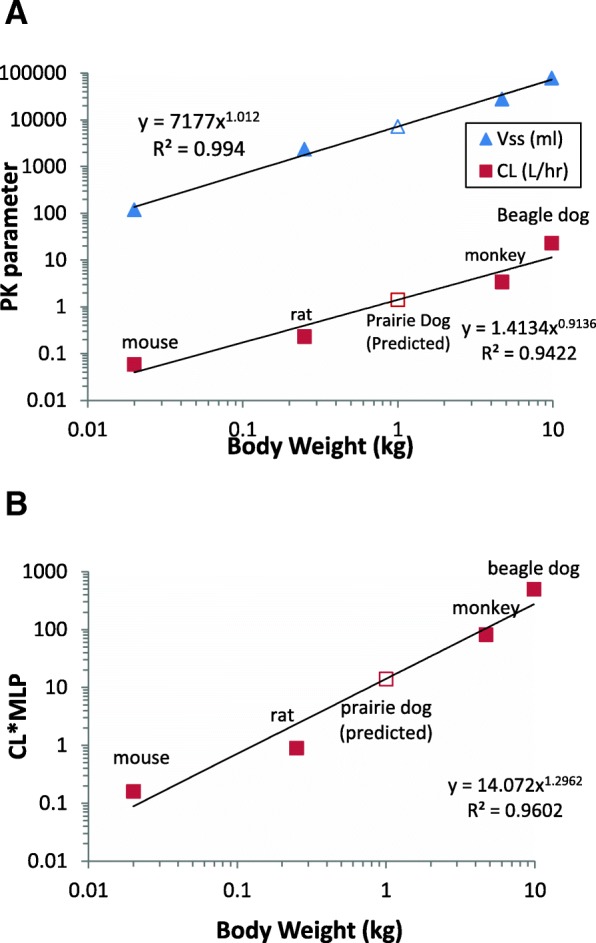
Allometric correlation plots of imatinib **a**) Simple allometry, (**b**) Simple allometry with ROE correction. The solid triangle symbol (▲) represents observed volume of distribution and solid square symbol (■) represents observed clearance. The open symbols represent predicted values.

Using the proportionality equations by allometric approaches as described in the methods section, we assessed the usefulness of interspecies scaling to predict PK parameters in prairie dogs, a species that was not previously employed in PK studies of TKIs. Interspecies scaling using data from four preclinical species indicated a correlation between nilotinib PK parameters (CL or V_ss_) and body weight (R^2^ > 0.9) with and without correction for plasma protein binding. The exponent of CL correlation plot was 1.13, and V_ss_ correlation plot was 1.12. After simple allometry, nilotinib CL in preclinical species was corrected with brain weight (BrW) to predict CL in and prairie dogs by the rule of exponents (ROE). The allometry plots are shown in Fig. 4. The predicted prairie dog CL, V_ss_ and prediction errors by these methods are listed in Table 3. The predicted prairie dog CL, V_ss_ and prediction errors by these methods are listed in Table 4. Fold error in prairie dog CL prediction was 2.24 to 2.5-fold, whereas fold error in V_ss_ prediction was under 2-fold. For imatinib, interspecies scaling using data from four preclinical species indicated a correlation between PK parameters (CL or V_ss_) and body weight (R^2^ > 0.9). The exponent of imatinib CL correlation plot was 0.91 indicating that MLP correction is needed for CL prediction, as per ROE. The exponent of imatinib V_ss_ correlation was 1.01. The allometry plots are shown in Fig. 5. The fold error in predicted prairie dog CL ranged from 1.07 to 2.24-fold of the observed value. The predicted V_ss_ of imatinib in prairie dogs was greater about 2.25 fold when simple allometry corrected for unbound plasma protein fraction was employed.

**Table 3:** CL and V_ss_ prediction of nilotinib in prairie dogs

S. No.	Method	Predicted Value	Fold Error
CL(L/hr./kg)			
1	SA (CL vs. BW)	0.42	2.24
2	SA (CL/fu_p_ vs. BW)	0.47	2.5
3	ROE (CL × BrW vs. BW)	0.44	2.32
V_ss_ (L/kg)			
1	SA (V_ss_ vs. BW)	1.45	1.25
2	SA (V_ss_/fu_p_ vs. BW)	1.62	1.40

*SA* simple allometry, *ROE* rule of exponents, *SSS* single species scaling

**Table 4:** CL and V_ss_ prediction of imatinib in prairie dogs

S. No.	Method	Value	Fold error
CL (L/hr./kg)			
1	SA (CL vs. BW)	1.41	1.78
2	SA (CL/fu_p_ vs. BW)	0.85	1.07
3	ROE (CL × MLP vs. BW)	1.78	2.24
V_ss_ (L/kg)			
1	SA (V_ss_ vs. BW)	7.18	4.43
2	SA (V_ss_/fu_p_ vs. BW)	3.65	2.25

*SA* simple allometry, *ROE* rule of exponents, *SSS* single species scaling

## Discussion

Clinical approval of antiviral drugs/biologics as potential countermeasures to some highly lethal viral pathogens is not akin to the approval process in other therapeutic areas either because there are not reliable sources of patients available for clinical trials or because it would be unethical to infect humans to conduct clinical trials. In these situations, by USFDA’s ‘Animal Efficacy Rule,’ first issued in 2002, the regulatory approval is based on demonstration of efficacy in appropriate animal models and the utilization of these models to construct PK assessments to support the human dose and the course of therapy [4]. The first drug approved under Animal Rule was pyridostigmine bromide, which is indicated for use after exposure to a nerve agent, Soman. The first biologic approved under this rule was raxibacumab, a monoclonal antibody intended to treat anthrax. To date, 12 products have been approved utilizing the Animal Rule, with more than half of them in the last three-four years, while several others such as the antiviral agents, tecovirimat (ST-246) and brincidofovir (CMX001) are under development indicating the increasing utility of this regulatory pathway [20–22].

Recent evidence indicates that TKIs, primarily developed as targeted anti-cancer drugs, exhibit antiviral activity, which is appealing in the context of their potential use as countermeasures against orthopoxviruses such as variola and monkeypox viruses [1]. Thus, in this study, we sought to characterize the PK of TKIs in various animal models to facilitate appropriate species selection for efficacy studies under the Animal Rule. A major pre-requisite for conducting non-clinical efficacy trials is to determine appropriate dosing regimens that would result in systemic exposure obtained clinically. However, these studies may use animal species, such as the prairie dog, that are necessary because of the specific viral model needed for efficacy testing. Since such animal models are not routinely used in early drug development stage, PK studies, as well as modification in the formulation due to inter-species physiological differences are often warranted before the efficacy assessments for FDA approval. Here approaches such as allometry based inter-species scaling, that are typically used for predicting human PK as an aid to first-in-human dose determination, may also be used to gain some insights a priori into clearance and V_ss_. Thus, as an overall secondary objective, we tested the predictability of PK data in animal models such as prairie dogs, heretofore not utilized for drug development, by interpolation of PK data across animal species.

Small animal models employed in our PK studies included C57BL/6 mice and guinea pigs. As indicated earlier, the oral bioavailability of these two drugs in C57BL/6 mice was quite high (50 and 100% for nilotinib and imatinib, respectively). However, the elimination half-lives were quite short (1–2 h). Thus, further testing of these agents in C57BL/6 mice is feasible but may require a continuous delivery system such as an Alzet® mini pump. Likewise, the elimination half-lives of these two compounds in guinea pigs were also quite short and oral bioavailability was poor. The reasons for the observed low oral bioavailability following extravascular dosing in guinea pigs are not apparent but may be a result of either incomplete absorption from the suspension formulation used and/or or extensive hepatic first pass metabolism in these species. Previously published data from studies employing CD-1 mice and Wistar-Hannover rats suggest that nilotinib is a low blood clearance compound in rodents as the systemic clearance only accounted for less than 25% of the hepatic blood flow (CL/Q_H_ = hepatic extraction ratio, CD-mice: 6.7%; Wistar-Hannover rats: 10.0%) [13]. This suggests that the contribution of hepatic first-pass metabolism to the observed poor oral bioavailability is likely to be low. Nilotinib is a drug with low water solubility and poor to moderate permeability and as such it can be considered as a Biopharmaceutics Classification System class II/IV compound. In fact, niliotinib exhibits pH-dependent solubility and has on oral absorption of 30% in the fasted state in humans. At fed state, the absorption drastically increases probably due to mechanisms such as increased solubility in the presence of bile salts and longer gastric emptying time. Thus, solubility limited absorption may be a primary factor restricting the oral bioavailability of the drug. Overall, it appears that the use of small rodents for anti-viral efficacy testing may be limited due to unfavorable PK properties such as poor oral availability and/or short elimination half- life.

The prairie dog is another rodent surrogate system for studying human orthopoxviruses [9] due to their high susceptibility to monkeypox virus via multiple routes such as intradermal [23] intranasal, [24] and intraperitoneal [25]. In single-oral dose PK study, mean terminal half-life of nilotinib was 3.5 h. or 7.5 h depending on the formulation, whereas the mean half-life of imatinib was 2.2 h. Bioavailability of both drugs in prairie dogs was similar when prepared in NMP/PEG 300. However, nilotinib exhibited lower oral bioavailability when prepared as Avicel/HPMC suspension formulation compared to soluble NMP/PEG 300 formulation. Large intra-species variability in plasma levels of both drugs in prairie dogs was possibly due to the outbred nature, wild-caught source, and genetic variability. The longer half-life of nilotinib in prairie dog makes it a suitable larger rodent model for conducting multiple dose PK and efficacy assessments.

Single dose PK of nilotinib was also investigated in Cynomolgus monkeys, which serve as a large animal non-rodent species for antiviral drug testing. This study was performed to find the systemic drug levels and bioavailability upon administration of nilotinib suspension formulation and to design a dosage regimen for conducting subsequent tolerability studies. The oral terminal half-life of nilotinib in monkeys was found to be 5.2 h compared to 7.8 h for intravenous administration. Oral bioavailability was estimated to be 13%. Overall, these PK observations are consistent with an earlier report by Xia et al. [13], employing a different oral and intravenous formulation. Following intravenous dose, nilotinib half-life and V_ss_ in monkeys in our studies were higher than the Xia et al. study possibly due to differences in the formulation.

Another impact of the aforementioned limited and pH-dependent aqueous solubility of nilotinib was the need to modify the formulation we employed during the course of this study. Nilotinib, while soluble in an acidic environment, is poorly soluble at pH above 4.5 [13]. There is a lack of suitable intravenous nilotinib formulation in humans. The formulation used in previously reported PK studies have varied based on the animal model. Xia et al. employed 0.5% HPMC suspension for oral PK studies in CD-1 mice, rats, beagle dogs, and monkeys while for intravenous formulation, nilotinib was prepared in cremophor:dimethylacetamide:5% dextrose (20:10:70, *v*/v/v). For their intravenous PK study in dogs, Solutol® HS 15 was used instead of cremophor [13]. In our oral single dose PK studies, we initially employed a formulation of nilotinib/NMP (20 mg/ml) in PEG 300 (1:10). However, in subsequent tolerability studies, toxicity such as bone marrow suppression was noticeable even in the vehicle-treated mice, which was attributable to NMP co-solvent used (D.K., data not shown). Further, this formulation was not tolerated in prairie dog multi-dose studies (J.S., data not shown) with side effects such as weight loss, severe diarrhea, and elevated liver enzymes in both drug formulation treated and vehicle-treated animals. Hence, the formulation was modified for all further prairie dog and mouse studies, along with studies in guinea pigs and monkeys to an oral suspension consisting of Avicel®-RC 591 and HPMC. This formulation was found to be tolerable for multiple dose PK studies in prairie dogs.

One limitation in our studies is that the experiments in nilotinib and imatinib are not balanced since imatinib experiments involved only two species. However, our findings add to the existing information on the PK of this drug by providing insights into its disposition in animal models not employed heretofore. PK results in these preclinical species are now being utilized for designing dosage regimes to simulate human-relevant systemic exposure upon single and multiple dose studies and facilitate antiviral efficacy assessments. As indicated earlier, for chronic dosing C57BL/6 mice may be used if these two drugs are provided via a continuous input mechanism in order to to deliver doses sufficient to counter poxvirus infections. To achieve a human-relevant steady state nilotinib concentration of around 1000 ng/ml in prairie dogs and monkeys, a twice-daily oral dosage regimen is being employed in further studies for antiviral efficacy testing.

As a secondary objective, we evaluated if PK data from previously published animal studies can be utilized to predict PK of nilotinib and imatinib in previously untested species such as prairie dogs, using the allometric approach of inter-species scaling. To this end, allometric correlation of PK parameters (CL and V_ss_) with body weight was performed utilizing previously reported CL and V_ss_ values in other species. There was a good correlation between CL and V_ss_ with the body weight (R^2^ > 0.9) among the four preclinical species used. For nilotinib, interspecies scaling indicated that the fold error in prairie dog CL prediction was greater than 2-fold whereas fold error in prairie dog V_ss_ prediction was under 2-fold. While imatinib CL prediction in prairie dogs was within 2-fold and about 2.25-fold for V_ss_ when using simple allometry method with fraction unbound to plasma protein correction. Thus, it appears that the allometry approaches represent a good starting point and provide preliminary insights in predicting PK parameters and designing dosage regimen in heretofore untested species to facilitate Animal Rule. However, they may not substitute initial dose-finding PK studies due to associated prediction errors attributable to inter-species and intra-species variability in drug disposition. The limitations are largely due to the empirical nature of the allometric approaches which do not incorporate physiological differences across species.

## Conclusions

In summary, pharmacokinetic studies were conducted to facilitate the use of Animal Rule for the potential repurposing of TKIs, nilotinib and imatinib, as antiviral agents. Based on the overall oral bioavailability and systemic exposure achieved, prairie dogs and monkeys may be suitable rodent and non-rodent species to perform further efficacy testing of TKIs against orthopoxvirus infections. Although rodents such as mice and guinea pigs represent an important tool for initial antiviral efficacy testing of TKIs, inadequate PK attributes such as short half-life and/or low oral bioavailability may limit their utility for further PK-PD investigations. Allometry-based inter-species interpolation of data appears to be an useful tool for a priori initial prediction of PK parameters in animal species not tested heretofore.

## Abbreviations

TKIs: Tyrosine kinase inhibitors
PK: Pharmacokinetics
PK-PD: Pharmacokinetics-pharmacodynamics
MCMs: Medical countermeasures
CL: Clearance
V: Volume of distribution
AUC: Area under the curve
BW: Body weight
Cmax: Maximum plasma concentration
SA: Simple allometry
ROE: Rule of exponents

## References

[1] Reeves PM, Smith SK, Olson VA, Thorne SH, Bornmann W, Damon IK, Kalman D (2011). Variola and monkeypox viruses utilize conserved mechanisms of virion motility and release that depend on abl and SRC family tyrosine kinases. J Virol.

[2] Reeves PM, Bommarius B, Lebeis S, McNulty S, Christensen J, Swimm A, Chahroudi A, Chavan R, Feinberg MB, Veach D (2005). Disabling poxvirus pathogenesis by inhibition of Abl-family tyrosine kinases. Nat Med.

[3] McFadden G (2005). Gleevec casts a pox on poxviruses. Nat Med.

[4] Snoy PJ (2010). Establishing efficacy of human products using animals: the US food and drug administration's "animal rule**"**. Vet Pathol.

[5] Hutson CL, Damon IK (2010). Monkeypox virus infections in small animal models for evaluation of anti-poxvirus agents. Viruses.

[6] Smee DF (2008). Progress in the discovery of compounds inhibiting orthopoxviruses in animal models. Antivir Chem Chemother.

[7] Connolly BM, Steele KE, Davis KJ, Geisbert TW, Kell WM, Jaax NK, Jahrling PB (1999). Pathogenesis of experimental Ebola virus infection in Guinea pigs. J Infect Dis.

[8] Americo JL, Moss B, Earl PL (2010). Identification of wild-derived inbred mouse strains highly susceptible to monkeypox virus infection for use as small animal models. J Virol.

[9] Smith SK, Self J, Weiss S, Carroll D, Braden Z, Regnery RL, Davidson W, Jordan R, Hruby DE, Damon IK (2011). Effective antiviral treatment of systemic orthopoxvirus disease: ST-246 treatment of prairie dogs infected with monkeypox virus. J Virol.

[10] Golden JW, Josleyn M, Mucker EM, Hung CF, Loudon PT, Wu TC, Hooper JW (2012). Side-by-side comparison of gene-based smallpox vaccine with MVA in nonhuman primates. PLoS One.

[11] Mucker EM, Goff AJ, Shamblin JD, Grosenbach DW, Damon IK, Mehal JM, Holman RC, Carroll D, Gallardo N, Olson VA (2013). Efficacy of tecovirimat (ST-246) in nonhuman primates infected with variola virus (smallpox). Antimicrob Agents Chemother.

[12] Shurtleff AC, Bavari S (2015). Animal models for ebolavirus countermeasures discovery: what defines a useful model?. Expert Opin Drug Discov.

[13] Xia B, Heimbach T, He H, Lin TH (2012). Nilotinib preclinical pharmacokinetics and practical application toward clinical projections of oral absorption and systemic availability. Biopharm Drug Dispos.

[14] Oostendorp RL, Buckle T, Beijnen JH, van Tellingen O, Schellens JH (2009). The effect of P-gp (Mdr1a/1b), BCRP (Bcrp1) and P-gp/BCRP inhibitors on the in vivo absorption, distribution, metabolism and excretion of imatinib. Investig New Drugs.

[15] Gupta B, Poudel BK, Tran TH, Pradhan R, Cho HJ, Jeong JH, Shin BS, Choi HG, Yong CS, Kim JO (2015). Modulation of pharmacokinetic and cytotoxicity profile of Imatinib Base by employing optimized nanostructured lipid carriers. Pharm Res.

[16] Neville K, Parise RA, Thompson P, Aleksic A, Egorin MJ, Balis FM, McGuffey L, McCully C, Berg SL, Blaney SM (2004). Plasma and cerebrospinal fluid pharmacokinetics of imatinib after administration to nonhuman primates. Clin Cancer Res.

[17] Ishizuka M, Nagai S, Sakamoto KQ, Fujita S (2007). Plasma pharmacokinetics and CYP3A12-dependent metabolism of c-kit inhibitor imatinib in dogs. Xenobiotica.

[18] Jones RD, Jones HM, Rowland M, Gibson CR, Yates JW, Chien JY, Ring BJ, Adkison KK, Ku MS, He H (2011). PhRMA CPCDC initiative on predictive models of human pharmacokinetics, part 2: comparative assessment of prediction methods of human volume of distribution. J Pharm Sci.

[19] Kretz O, Weiss HM, Schumacher MM, Gross G (2004). In vitro blood distribution and plasma protein binding of the tyrosine kinase inhibitor imatinib and its active metabolite, CGP74588, in rat, mouse, dog, monkey, healthy humans and patients with acute lymphatic leukaemia. Br J Clin Pharmacol.

[20] Park GD, Mitchel JT (2016). Working with the U.S. Food and Drug Administration to obtain approval of products under the animal rule. Ann N Y Acad Sci.

[21] Leeds JM, Fenneteau F, Gosselin NH, Mouksassi MS, Kassir N, Marier JF, Chen Y, Grosenbach D, Frimm AE, Honeychurch KM (2013). Pharmacokinetic and pharmacodynamic modeling to determine the dose of ST-246 to protect against smallpox in humans. Antimicrob Agents Chemother.

[22] Trost LC, Rose ML, Khouri J, Keilholz L, Long J, Godin SJ, Foster SA (2015). The efficacy and pharmacokinetics of brincidofovir for the treatment of lethal rabbitpox virus infection: a model of smallpox disease. Antivir Res.

[23] Hutson CL, Olson VA, Carroll DS, Abel JA, Hughes CM, Braden ZH, Weiss S, Self J, Osorio JE, Hudson PN (2009). A prairie dog animal model of systemic orthopoxvirus disease using west African and Congo Basin strains of monkeypox virus. J Gen Virol.

[24] Hutson CL, Carroll DS, Self J, Weiss S, Hughes CM, Braden Z, Olson VA, Smith SK, Karem KL, Regnery RL, Damon IK (2010). Dosage comparison of Congo Basin and west African strains of monkeypox virus using a prairie dog animal model of systemic orthopoxvirus disease. Virology.

[25] Xiao SY, Sbrana E, Watts DM, Siirin M, da Rosa AP, Tesh RB (2005). Experimental infection of prairie dogs with monkeypox virus. Emerg Infect Dis.

